# Strategies for Preparation of Chiral Stationary Phases: Progress on Coating and Immobilization Methods

**DOI:** 10.3390/molecules26185477

**Published:** 2021-09-09

**Authors:** Carla Fernandes, Joana Teixeira, Madalena M. M. Pinto, Maria Elizabeth Tiritan

**Affiliations:** 1 Laboratório de Química Orgânica e Farmacêutica, Departamento de Ciências Químicas, Faculdade de Farmácia da Universidade do Porto, Rua Jorge de Viterbo Ferreira 228, 4050-313 Porto, Portugal; jbteixeira@live.com.pt (J.T.); madalena@ff.up.pt (M.M.M.P.); elizabeth.tiritan@iucs.cespu.pt (M.E.T.); 2 Centro Interdisciplinar de Investigação Marinha e Ambiental (CIIMAR), Universidade do Porto, Terminal de Cruzeiros do Porto de Leixões, Avenida General Norton de Matos, s/n, 4450-208 Matosinhos, Portugal; 3 CESPU, Instituto de Investigação e Formação Avançada em Ciências e Tecnologias da Saúde, Rua Central de Gandra, 1317, 4585-116 Gandra, Portugal

**Keywords:** chiral selector, click chemistry, coupling agent, dynamic coating, intermolecular polycondensation, isocyanate derivatives, photochemical method, radical polymerization, thermal method

## Abstract

Enantioselective chromatography is one of the most used techniques for the separation and purification of enantiomers. The most important issue for a specific successful enantioseparation is the selection of the suitable chiral stationary phase (CSP). Different synthetic approaches have been applied for the preparation of CSPs, which embrace coating and immobilization methods. In addition to the classical and broadly applied coating and immobilization procedures, innovating strategies have been introduced recently. In this review, an overview of different methods for the preparation of coated and immobilized CSPs is described. Updated examples of CSPs associated with the various strategies are presented. Considering that after the preparation of a CSP its characterization is fundamental, the methods used for the characterization of all the described CSPs are emphasized.

## 1. Introduction

The separation of enantiomers in an analytical and preparative scale is undoubtedly a matter of crucial importance in diverse research areas [1]. Liquid chromatography (LC) using chiral stationary phases (CSPs) proved to be the most versatile and useful approach for the evaluation of enantiomeric purity in quality control [2,3], the preparative enantioresolution process [4,5], monitoring enantiomeric reactions [6,7], food analysis [8,9], the stereochemistry determination of natural compounds [10,11], pharmacokinetic [12] and environmental studies [13,14], among other applications. Over the years, several types of CSPs have been developed which comprise polysaccharide derivatives, Pirkle-type, cyclodextrins, crown-ether, proteins, macrocyclic antibiotics, ion-exchange, cyclofructans, synthetic polymers and molecularly imprinted CSPs [15,16,17].

To follow the constant challenges on different areas as well as the advances in chromatographic instrumentation and technical progress, the development of new CSPs for LC has been a continuous and evolutionary subject [15]. The strategies encompassed the development of new chiral selectors, the introduction of new chromatographic supports and the application of different synthetic approaches for the preparation of CSPs. The first described CSPs were prepared by coating the chiral selector onto the surface of porous silica by Davankov [18]. In that time, this achievement was remarkable since it settled a background for further applications. The advantages inherent to the coating approach include a fast and simple process for the preparation of the CSP and the manageable optimization of the loading selector [19]. Since specific requirements for the chromatographic support are not necessary, coated CSPs are recognised by their reproducibility [20]. Nevertheless, coated-type CSPs also have drawbacks such as incompatibility with many organic solvents used as the mobile phase. Additionally, the swelling of the selector can occur which limits the lifetime of the column [20,21]. These critical points of the coated-CSPs triggered the search for other strategies. Since the report of the first immobilized Pirkle-type CSP [22], this strategy proved to be a feasible alternative. Immobilized CSPs present a superior resistance for a broader variety of organic solvents which contributed to an expansion of the enantioseparation applications [21].

**Figure 1:**
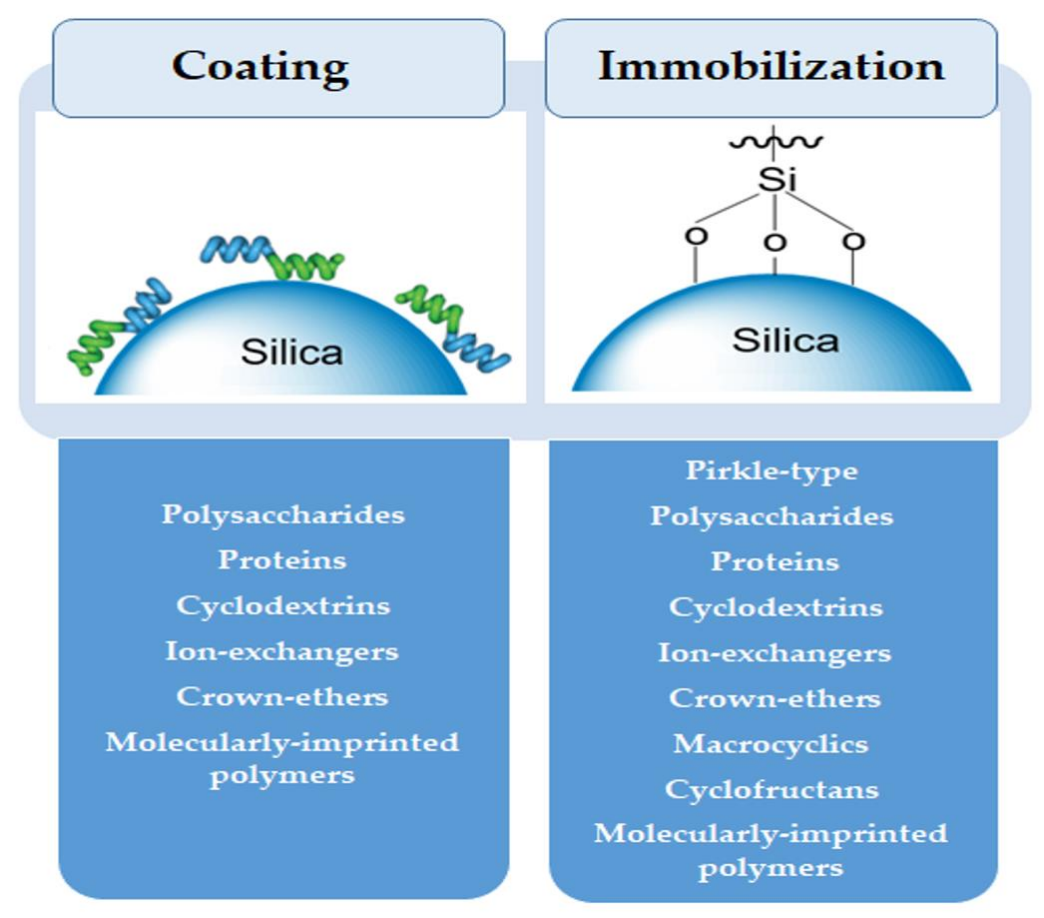
Types of chiral selectors typically coated or immobilized onto the chromatographic support.

In fact, according to the type of chiral selector, immobilization is preferred to the coating method (Figure 1). Nevertheless, for some types of CSPs, such as protein and polysaccharide-based ones, immobilized CSPs could present a reduction in the chiral recognition ability as a consequence of the immobilization process [23]. To minimize this drawback, some considerations should be taken into account such as the control of the native support or chiral selector derivatization as well as the degree of immobilization, to preserve the structure of the chiral selector. Over the last years, diverse immobilization strategies have been introduced which proved to be efficient and do not considerably interfere with the chiral recognition ability of the selector.

It is important to point out that coated CSPs continue to be one of the most applied approaches in enantioselective LC, also representing an important percentage of the commercialized CSPs [24,25]. The continuous success of some coated CSPs could be attributed mainly to a superior chiral recognition ability compared with immobilized CSPs [26]. Recently, different immobilization synthetic strategies were introduced in order to simplify the immobilization process, and to meet the green chemistry principles, in addition to other goals.

In this review, different methods for the preparation of coated and immobilized CSPs are described and the progresses on coating and immobilization methods are focused. In addition, updated examples of CSPs associated with the different strategies are presented and the methods used for their characterization are also emphasised.

## 2. Coating Method

One of the first types of coated CSPs were the polysaccharide-based ones, which were described by Okamoto et al. [27] using macoporous silica. Later on, the advantage of the meso (500 Å) or microporous (120 Å) silica as the support for the carbohydrate carbamate silica was reported [19,28,29], including the application in preparative enantioseparation [30]. Coated CSPs are obtained from a solution of the chiral selector mixed with the chromatographic support, typically silica or derivatized silica (e.g., aminopropyl silica) by gently stirring and evaporation of the organic solvent [19,30]. During the coating process, the porous structure of the silica should be maintained. The coating process could also be performed, for example, with inorganic oxides instead of silica particles. Alumina, zirconia and titania were selected since they present a superior stability to a wide range of pH values than silica. Similar to silica, a polymer could be coated on particles of these oxides [31,32]. Polymer-coated metal oxides exhibited a good pH stability and chromatographic selectivity, and allowed a remarkable column performance and lifetime [33,34,35]. Nevertheless, the reduced commercial availability of metal-oxide particles prevented the generalized use of these materials.

**Figure 2:**
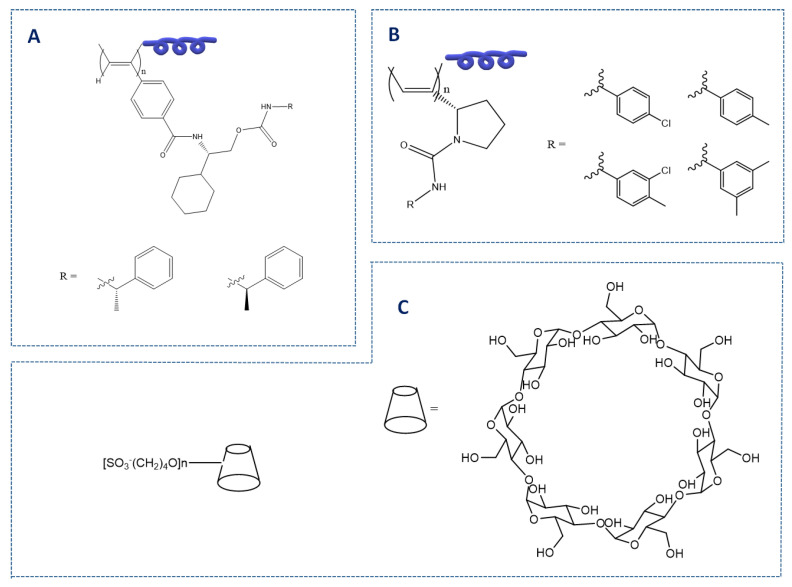
Recent selectors (**A**–**C**) for chiral stationary phases prepared by coating method.

Recently, two polymeric-coated CSPs based on helical poly(phenylacetylene) derivatives were prepared by Okamoto et col. [36] (Figure 2A). The solvent for the coating procedure was a mixture of trifluoroacetic acid and tetrahydrofuran being the derivatives coated on aminopropyl-silanized silica gel. The new CSPs presented a noteworthy solvent tolerability being compatible with chloroform and tetrahydrofuran, which are solvents typically not tolerate by coated-type CSPs. Nuclear magnetic resonance (NMR) was applied for the characterization of the obtained CSPs [36].

In another example, a new CSP was prepared by Li et al. [37] by coating cellulose (3,5-dimethylphenylcarbamate) onto reduced graphene oxide which was covalently coupled to microspheres of silica gel. The new CSP presented a superior enantioselectivity performance when compared with classical columns comprising the same cellulose derivative coated onto silica. CSP characterization was accomplished through an elemental analysis, transmission electron microscopy (TEM), Fourier-transform infrared spectroscopy (FTIR) and thermogravimetric analysis (TGA) [37].

New derivatives of chitosan with isopropylthiourea and carbamate groups were coated onto 3-aminopropyl silanized silica gel by Okamoto et col. [38]. Comparing the enantioseparation performance of chitosan 2-isopropylthiourea derivatives with the commercial CSP CHIRALCEL OD^®^ and chitosan derivatives with substituents at position 2, the new CSPs demonstrated a higher enantioselectivity and resolution for some racemates. CSPs were characterized by NMR, FTIR, elemental analysis and TGA [38].

Shi et al. [39] prepared CSPs based on proline-derived helical poly(phenylacetylene)s (Figure 2B) coated on porous silica gel. This type of CSPs presented an advantage of adjusting its resolution ability according with the coating solvent. Coating solvents influenced the chiral recognition by interfering with the conformation of the helicity of the polymer. For some racemates, the new CSP demonstrated an improved enantioselectivity than the commercials columns CHIRALCEL OD-H^®^ and CHIRALPAK AD-H^®^ under the normal-phase elution mode. CSPs characterization was performed by NMR, elemental analysis and Raman measurement [39].

Nowadays, in addition to the classical method, there are other approaches for the coating process to develop new CSPs. A recent example was described by Cong et al. [40], who first sulfonated porous poly(styrene-divinylbenzene) microparticles being the chitosan coated onto this chromatographic support. The sulfonated microparticles were added to an aqueous solution of chitosan proceeded by a washing and a cure step with ultraviolet (UV) radiation (seed swelling seed polymerization method). The back pressure of this new CSP was reduced even with a high flow rate of the mobile phase. The CSP was characterized by scanning-electron microscope (SEM), adsorption–desorption isotherms of N_2_ and its surface area was obtained through the Brunauer–Emmett–Teller (BET) theory [40].

More recently, the application of dynamic coating, an in situ technique, for the preparation of CSPs was described [41]. This procedure consists of the injection of a mobile phase saturated with the CSP into the column. The concentration of the CSP in the solution determines the thickness of the stationary phase film [41]. Dynamic coating allows the replacement of the selector being one of the benefits for this technique [42].

Folprechtová et al. [43] prepared by dynamic coating three new CSPs based on sulfobutylether-β-cyclodextrin derivatives (Figure 2C), with different degrees of substitution. The new CSPs presented a great enantioselectivity. It was found that a superior degree of substitution resulted in a higher enantioresolution performance. CSPs characterization was performed by a gravimetric analysis [43].

Another example of a dynamic coating procedure was described by Naghdi et al. [42], specifically, of coating hydroxypropyl-β-cyclodextrin onto porous silica. The new CSP was prepared in a pillar array column, a feasible alternative to packed bed columns and monoliths since they allowed a homogeneous flow direction and a reduced back pressure. In terms of chromatographic performance, the new CSP demonstrated an improved enantioresolution ability, for some racemates, when compared with the equivalent commercial column CHIRALPAK AD-RH^®^. SEM was used for the characterization of the CSP [42].

CSPs comprising proteins were also prepared by a coating technique based on multiple interactions, such as Van der Waals, ionic and hydrogen bonding, between the chromatographic support and the protein [44]. Despite silica being the most common chromatographic support [45], some metals could also be applied (titanium, aluminium or zirconium) [44]. These materials presented a great mechanical strength although their affinity for some proteins restricted their application [44]. Synthetic polymers are widely used for protein coating since they allow the adjustment of the reaction conditions according to the protein. Polymers also present a considerable thermal and chemical resistance and their synthesis can be considered simple in general [44]. To guarantee the function of the protein as chiral selector, the coating procedure should not be aggressive. The stability of the column can be compromised since interactions between the selector and chromatographic support are vulnerable [46]. To perform the selector coating, it is necessary to have suitable functional groups on the surface of both the protein and chromatographic support [44]. Surface-modifying agents frequently applied for the functionalization of the chromatographic support are based on derivatives of amino-, diol- and epoxide groups [46].

**Figure 3:**
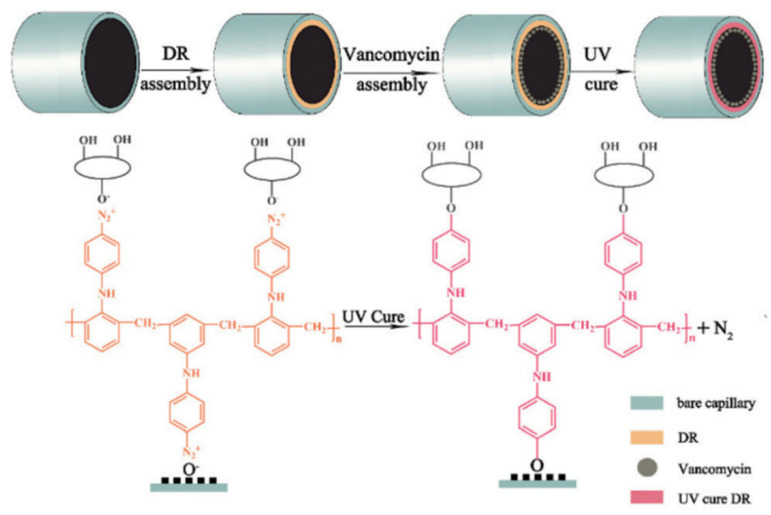
Preparation of the diazo resin (DR)/vancomycin coatings on the inner wall of the column (reprinted from Publication Xing, J.; Wang, F.; Cong, H.; Wang, S.; Shen, Y.; Yu, B., *Analysis of proteins and chiral drugs based on vancomycin covalent capillary electrophoretic coating. Analyst*. 2021, 146, (4), 1320–1325. Copyright © 2021, with permission from Royal Society of Chemistry [47]).

The coating of a protein on the inner wall of a capillary column has recently been described by Xing et al. [47]. They used a photosensitive diazo resin (DR) as the coupling agent, and vancomycin was coated on the inner wall of the column (Figure 3) [47]. The vancomycin-based coated column proved to be resistant to protein adsorption and chiral separation to a certain extent. Efficient separations of a mixture of lysozyme, bovine serum albumin, myoglobin and ribonuclease A were achieved. In addition, promethazine was also successfully enantioseparated. The coating process was monitored by UV–Vis. Atomic force microscopy (AFM) was performed for a capillary surface morphology analysis [47].

## 3. Immobilization Method

The immobilization process between a chiral selector and a chromatographic support can be performed using different synthetic strategies which include classical methods, namely, immobilization using isocyanates, coupling agents, radical polymerization, intermolecular polycondensation, or more recent methodologies, such as click chemistry, photochemical and thermal methods.

### 3.1. Immobilization Based on Isocyanate Derivatives

Isocyanates were applied to synthetize derivatives that could be used for the further immobilization of a chiral selector onto the chromatographic support [48]. The first immobilization of a polysaccharide derivative onto silica gel using a diisocyanate as a cross-linker was reported by Okamoto’s group in 1987 [49]. One disadvantage of this methodology was related with the non-regioselectivity of the isocyanate reagent. In excess, the diisocyanate could react with additional points of the selector leading to an extreme degree of immobilization reducing the enantioresolution ability [23].

In order to minimize disorders in the structure of the selector that could compromise the enantioresolution capacity, a new strategy for immobilization was introduced by Okamoto et col., in 1996 [50]. It was an attractive option since this technique allows a reduced number of chemical bonds (just at a terminal group of the chiral selector). For the immobilization of polysaccharide derivatives onto silica gel, an isocyanate derivative was also applied [50].

**Figure 4:**
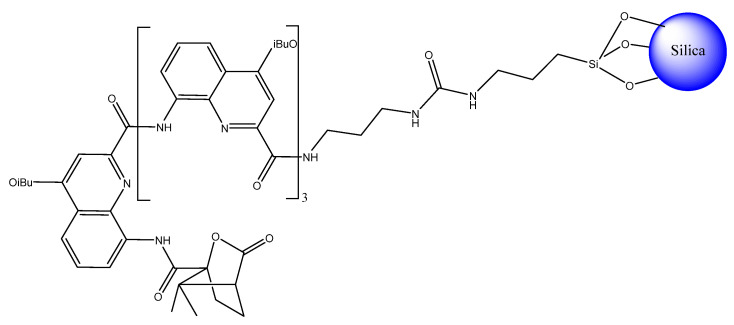
Chiral stationary phase comprising a *P*-helical quinoline oligoamide foldamer immobilized using an isocyanate derivative.

Recently, a *P*-helical quinoline oligoamide foldamer was immobilized onto silica by Noguchi et al. [51] (Figure 4). The *P*-helicity was promoted by an appended chiral moiety at the *N*-terminus. The immobilization onto silica was performed by the introduction of a trimethoxysilyl group at the opposing terminal position of the foldamer. The new CSP did not present a wide range of applicability although it demonstrated an enhanced enantioselectivity for compounds with a similar structure than the chiral selector. An elemental analysis and NMR were applied for CSP characterization [51].

**Scheme 1:**
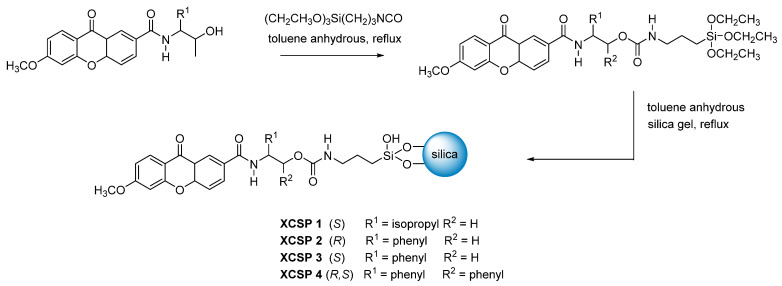
Synthesis of immobilized-chiral stationary phases based on chiral derivatives of xanthones (XCSPs) using an isocyanate derivative.

Our group recently prepared new CSPs comprising chiral derivatives of xanthones (CDXs) and the strategy used for binding the chiral selectors to the chromatographic support was through the synthesis of silylated derivatives by a reaction with 3-(triethoxysilyl)propyl isocyanate, allowing the covalent linkage to the silica (Scheme 1). The CSPs characterization was performed by elemental analysis [52,53]. The CDXs, in addition to interesting biological activities [54,55], showed enantioselectivity for chiral analytes, under multimodal elution conditions [52,53].

By using the same isocyanate derivative, 3-(trietoxysilyl)propyl isocyanate, Gasparrini et col. [56] recently developed teicoplanin and vancomycin-based CSPs immobilized onto sub 2 μm fully porous silica. The selector bonding was dependent of the proportion of the isocyanate linker, temperature and time of reaction. This classical method allowed to obtain macrocyclic-based CSPs suitable for UHPLC and capable of separating enantiomers from inorganic cations and anions. An improved enantioselectivity was found when compared with TeicoShell^®^ and VancoShell^®^ columns. The CSPs were characterized through an elemental analysis [56].

**Figure 5:**
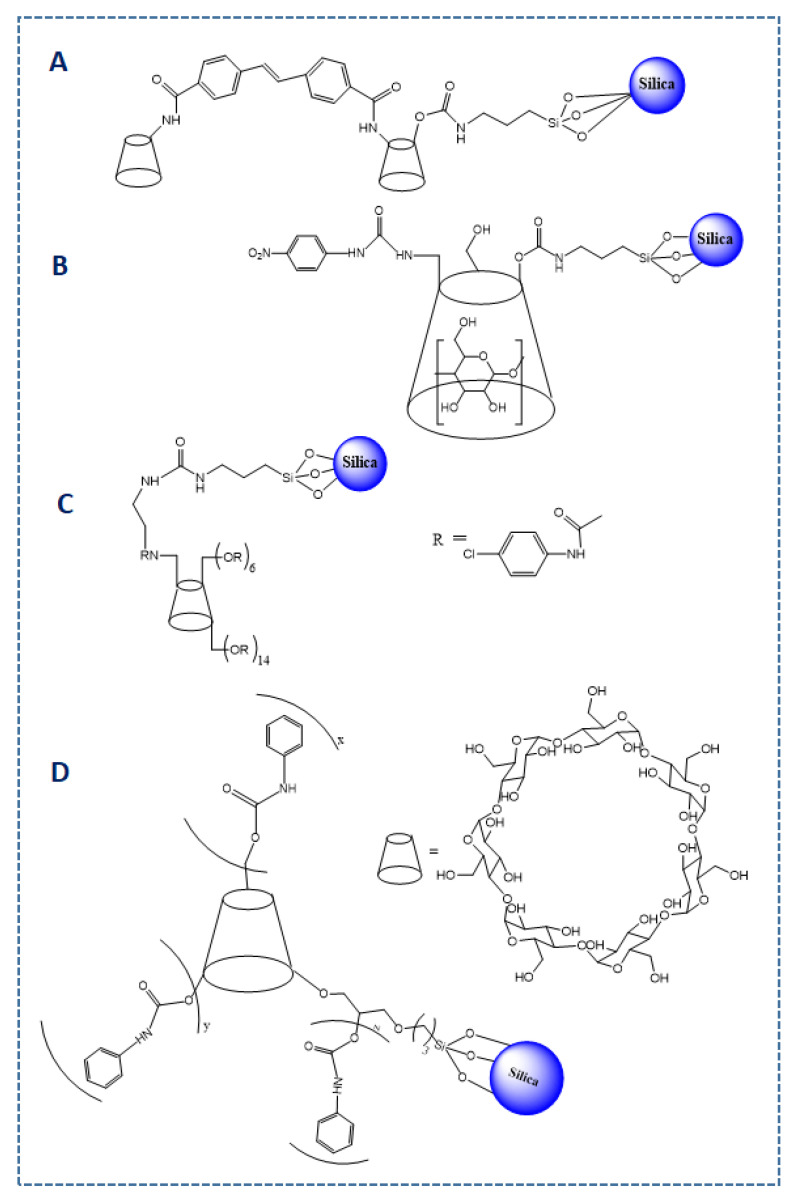
Cyclodextrin-chiral stationary phases (**A**–**D**) prepared using an isocyanate derivative.

Another example is the development of the 4,4-stilbene diamido-bridged bis(β-cyclodextrin)-bonded CSP (Figure 5A) by Shuang et al. [57]. They immobilized the chiral selector onto mesoporous silica gel after derivatization with an isocyanate derivative. An enhanced resolution with a reduced analysis time was achieved compared to the native β-cyclodextrin-based CSP. Mass spectrometry, NMR and FTIR were applied for the characterization of the developed CSP [57].

Shuang et al. [58] also developed a new β-cyclodextrin-bonded CSP based on a *mono*-6-amino-β-cyclodextrin immobilized onto ordered mesoporous silica gel (Figure 5B) using 3-(triethoxysilyl)propyl isocyanate. The introduction of an ureido moiety allowed the possibility to have a group with a hydrogen-bond accepting and donating properties. It also prevented hydrolysis usually associated with nitrobenzoate and nitrophenylcarbamate linkages. The new CSP demonstrated a superior enantioselectivity in comparison with native β-cyclodextrin CSP. Besides NMR, SEM, TEM, X-ray diffraction, N_2_ adsorptio–desorption isotherm, elemental analysis and TGA were applied on CSP characterization [58].

By using the same immobilization strategy, a *per*-4-chlorophenylcarbamate-β-cyclodextrin was immobilized onto silica gel just through one site by a one-step approach without a diazotization reagent by Sun et al. [59]. This CSP (Figure 5C) was compared with the commercial 3,5-dimethylphenyl carbamate-β-cyclodextrin-based CSP exhibiting a superior enantioselectivity due to improved interactions established with analytes. The new CSP was characterized by SEM, TEM, NMR, FTIR, elemental analysis and TGA [59].

A new cyclodextrin-based CSP was reported by Yi et al. [60] by using phenyl isocyanate to obtain the partially substituted phenylcarbamate-(3-(2-*O*-β-cyclodextrin)-2-hydroxypropoxy)-propylsilyl-appended silica particles (Figure 5D). The CSP demonstrated enantioselectivity for different positional isomers (nitrophenol and nitroaniline) and enantiomers of chiral drugs being operated in multi-mode LC mobile phase conditions. An improved enantioselectivity was obtained in comparison with commercial columns Cyclobond I DMP^®^ and Cyclobond I SN^®^. An elemental analysis and FTIR were applied for the characterization of this new CSP [60].

### 3.2. Immobilization Using Coupling Agents

The immobilization of chiral selectors onto the chromatographic support using coupling reagents was introduced in 1980 by Pirkle’s group [61]. They prepared new CSPs by linked D-3,5-dinitrobenzoylphenylglycine to aminopropyl silica using the coupling agent *N*-ethoxycarbonyl-2-ethoxy-l,2-dihydroquinoline (EEDQ) [61]. The main advantages associated with this immobilization method are the stability of the CSPs and the simplicity of the procedure; although the degree of racemization could compromise the results. Another drawback is the reduced yield in the coupling step [62]. Nevertheless, it is one of the most used method to prepared immobilized CSPs. It is commonly applied when aminopropyl silica is used as a chromatographic support being the carboxylic acid group typically presented on the chiral selector [63].

Recently, Agneeswari et al. [64] developed CSPs based on crown-ethers and the chiral unit 1-(1-naphthyl) using the same coupling agent (EEDQ). For all CSPs, the “matched” effect that occurs when the chiral recognition is potentiate by the cooperation between the two chiral units of diastereomeric chiral selectors was evaluated. It was inferred that the crown-ether unit could be successfully applied as a chiral tethering group. An elemental analysis and NMR were applied for CSP characterization [64].

**Scheme 2:**
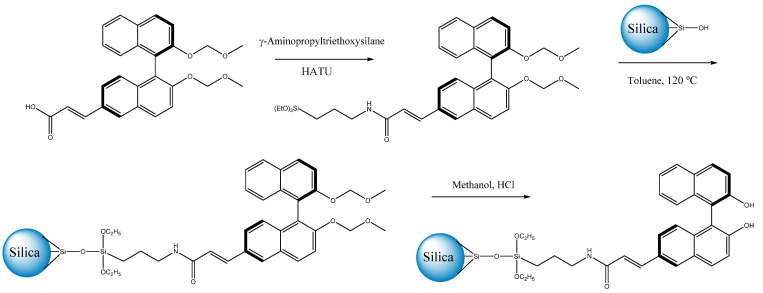
Synthetic route for immobilization of a chiral selector comprising a (*R*)-binaphthol unit using a coupling agent (HATU).

In addition to EEDQ, other coupling reagents were explored. A CSP comprising a (*R*)-binaphthol unit was prepared by Wang et al. [65] using the coupling reagent *O*-(7-azabenzotriazol-1-yl)-*N,N,N’,N’*-tetramethyluronium hexafluorophosphate (HATU) to synthetize an amide intermediate, from the acidic chiral selector and γ-aminopropyltriethoxysilane, and the further immobilization to silica (Scheme 2). The enantioseparation ability of this new CSP was evaluated under a normal elution mode. FTIR, SEM and elemental analysis allowed the characterization of this CSP [65].

**Scheme 3:**
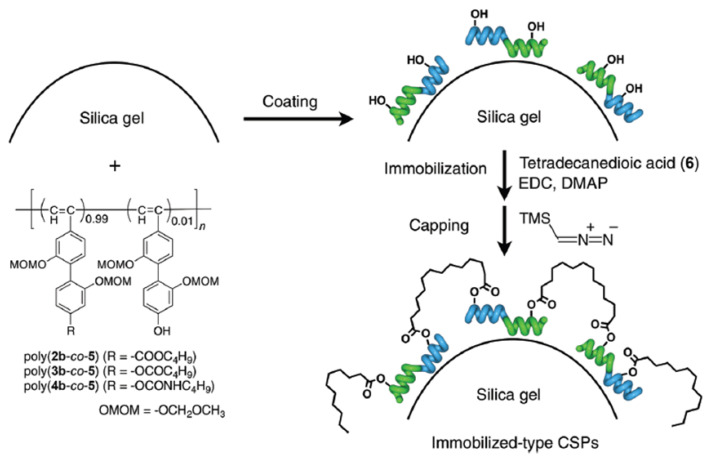
Immobilization of copolymers onto silica gel by cross-linking between hydroxyl groups of the copolymer chains coated onto silica gel with tetradecanedioic acid using a coupling agent (EDC) (reprinted from Publication Ishidate, R.; Sato, T.; Ikai, T.; Kanoh, S.; Yashima, E.; Maeda, K., *Helicity induction and memory effect in poly(biphenylylacetylene)s bearing various functional groups and their use as switchable chiral stationary phases for HPLC*. *Polym. Chem*. 2019, 10, (46), 6260–6268. Copyright © 2019, with permission from Royal Society of Chemistry [66]).

Maeda et col. [66] prepared new CSPs by the immobilization of copolymers based on poly(biphenylylacetylene)s (PBPAs) onto silica gel by cross-linking between hydroxyl groups of the copolymer chains coated onto silica gel with tetradecanedioic acid using 1-ethyl-3-(3-(dimethylamino)-propyl) carbodiimide (EDC) as a coupling agent (Scheme 3). This methodology induced helicity which could be switched through an alternated treatment with a solution containing the (*R*)- or (*S*)-enantiomer of a chiral alcohol. Some switchable CSPs demonstrated high enantioselectivity. NMR allowed the CSP’s characterization [66].

The same group also introduced new poly(diphenylacetylene)-based CSPs with optically active anilide pendants, which were bonded by using EDC as a coupling agent [67]. The presence of macromolecular helicity in the polymeric backbone induced during the synthesis of one CSP contributed towards an enhanced enantiorecognition ability. NMR, elemental analysis and TGA were applied for CSP characterization [67].

In another study, Ye et al. [68] linked bovine serum albumin on graphene quantum dots through a coupling reaction using the same coupling reagent (EDC). The original protein morphology changed after the adsorption on quantum dots exposing the chiral sites at the surface of the selector. The prepared CSP presented a recognisable biomolecular homochirality in the recognition of the analysed enantiomers. It was characterized by FTIR [68].

**Scheme 4:**
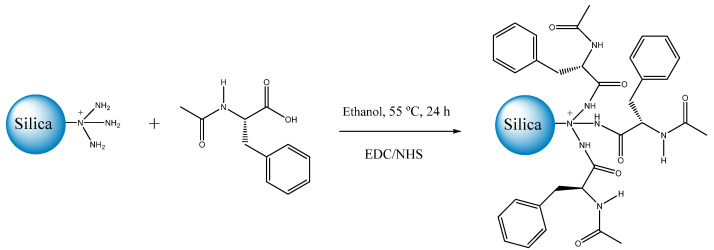
Immobilization of a chiral selector comprising poly(ethyleneimine) with *N*-acetyl-L-phenylalanine moieties using a coupling agent (EDC).

A new CSP based on poly(ethyleneimine) with *N*-acetyl-L-phenylalanine moieties were introduced by Wan et al. [69]. The chiral selector was immobilized onto silica gel using EDC and *N*-hydroxysuccinimide (NHS) (Scheme 4). The new CSP was able to enantioseparate warfarin, under the reversed-phase elution mode. FTIR, TGA and elemental analysis were applied for its characterization [69].

### 3.3. Immobilization by Radical Polymerization of Vinyl Groups

The immobilization of a cellulose derivative by radical polymerization was reported by Kimata et al. [70] in 1993. To improve the immobilization efficiency of the derivatives with vinyl groups, a vinyl monomer was introduced in the reaction mixture [71]. A copolymerization reaction occurred; however, if vinyl groups are introduced in excess, it could result in a decrease on enantioselectivity [48].

**Scheme 5:**
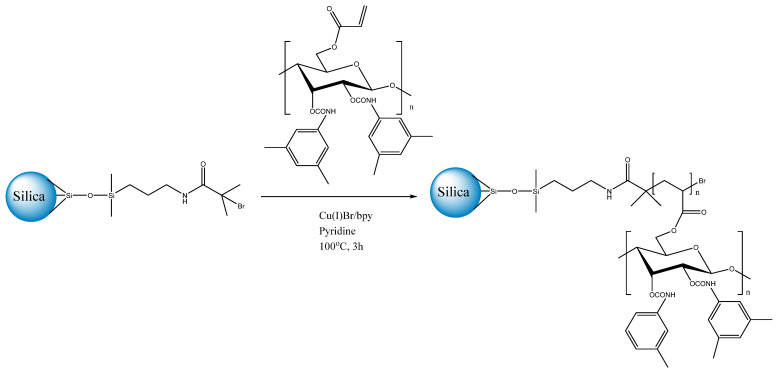
Immobilization of cellulose 2,3-bis(3,5-dimethylphenylcarbamate), having a polymerizable vinyl group, onto silica, by radical polymerization method.

Bae et al. [72] prepared a polysaccharide-immobilized CSP by a surface-initiated atom transfer radical polymerization of cellulose 2,3-bis(3,5-dimethylphenylcarbamate), having a polymerizable vinyl group, on the surface of a silica support (Scheme 5). It was found that the prepared CSP was very stable even when using tetrahydrofuran and chloroform in the mobile phase. Field-emission scanning electron microscopy, X-ray photoelectron spectroscopy, elemental analysis and thermal analysis were applied for the characterization of this CSP [72].

**Scheme 6:**
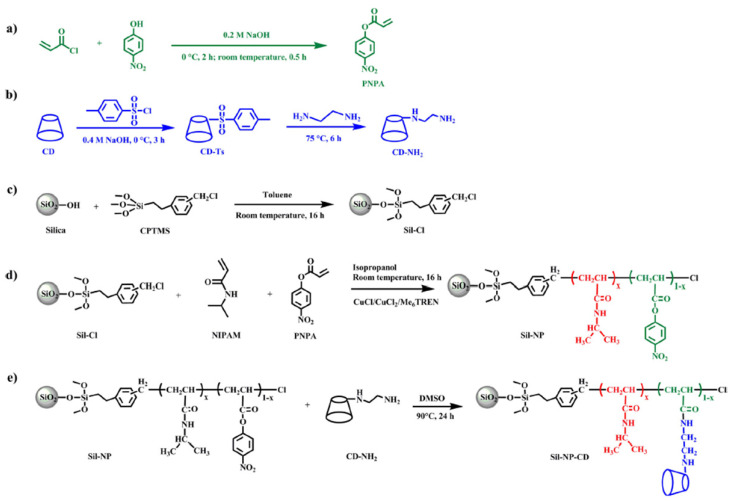
Synthesis route of PNPA (**a**), CD-NH_2_ (**b**), Sil-Cl (**c**), Sil-NP (**d**) and thermoresponsive chiral Sil-NP-CD stationary phase (**e**) (reprinted from Publication Ren, X.; Luo, Q.; Zhou, D.; Zhang, K.; Gao, D.; Fu, Q.; Liu, J.; Xia, Z.; Wang, D.L., Thermoresponsive chiral stationary phase functionalized with the copolymer of β-cyclodextrin and *N*-isopropylacrylamide for high performance liquid chromatography. *J. Chromatogr. A* 2020, 1618. Copyright © 2020, with permission from Elsevier [73]).

Inspired by the radical polymerization of the vinyl groups method, Ren et al. [73] developed a thermoresponsive CSP functionalized with a copolymer of β-cyclodextrin (Scheme 6). To trigger the synthesis of this new CSP, the *P*-nitrophenyl acrylate (PNPA) monomer was introduced in the reaction to copolymerize with the *N*-isopropylacrylamide (NIPAM) on the surface of the silica particles. A superior uniformization of the silica particles with a reduced size allowed an improvement in the column efficiency and peak shape. A promising performance for the enantioseparation of hydrophilic and hydrophobic compounds was achieved when comparing with the YMC CHIRAL β-CD BP^®^ column. For CSP characterization, NMR, X-ray photoelectron spectroscopy, elemental analysis, FTIR, TGA and SEM were performed [73].

### 3.4. Immobilization by Intermolecular Polycondensation

The strategy of immobilization by intermolecular polycondensation was reported more recently by Okamoto et col. [74]. The alkoxysilyl groups are known for their easy polymerization into polysiloxanes in acidic or basic media. Accordingly, this enhanced reactivity could be used for immobilization onto silica [23]. This technique allows the maintenance of the structure of the derivatives since the immobilization could be performed by using a small quantity of reagents, resulting in a reduced degree of cross-linking. Advantages inherent to this method include a similar recognition ability of the immobilized CSPs with the coated-type CSPs, the simplicity of the process, the immobilization efficiency and high degree of chiral recognition [75].

**Scheme 7:**
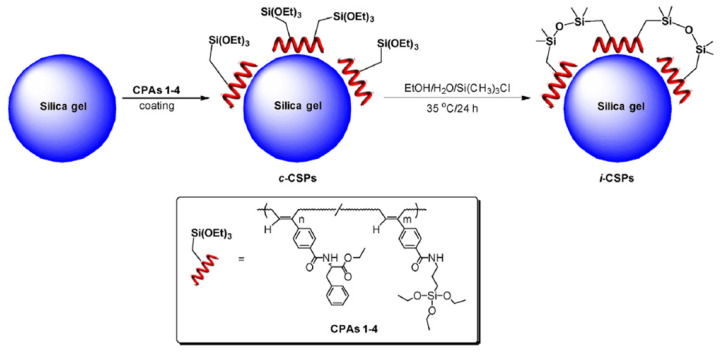
Immobilization of the copolymers (CPAs 1–4) on the silica gel by the intermolecular polycondensation of the triethoxysilyl groups (reprinted from Publication Zhou, Y.; Zhang, C.; Geng, Q.; Liu, L.; Dong, H.; Satoh, T.; Okamoto, Y., Immobilization of helical poly(phenylacetylene)s having *L*-phenylalanine ethyl ester pendants onto silica gel as chiral stationary phases for HPLC. *Polymer* 2017, 131, 17–24. Copyright © 2017, with permission from Elsevier [76]).

Zhou et al. [76] developed a new CSP based on helical poly(phenylacetylene)s comprising *L*-phenylalanine ethyl ester pendants immobilized onto silica gel by the intermolecular polycondensation of triethoxysilyl groups (Scheme 7). In this case, an enhanced enantiorecognition ability and solvent compatibility were achieved when compared with the correspondent coated CSP. The characterization of the CSP was performed by NMR and TGA [76].

Chen et al. [77] prepared 10 β-cyclodextrin derivatives bearing substituted carbamate or benzoate groups. The new β-cyclodextrin derivatives were immobilized onto silica gel by intermolecular polycondensation of triethoxysilyl groups. The new CSPs showed an enantiorecognition capacity similar to commercial immobilized derivatives of cellulose and amylose. The characterization of the CSPs was performed by NMR and TGA [77].

### 3.5. Covalent Linkage by Click Chemistry

Click chemistry, introduced in 2001 by Sharpless et col. [78], concerns reactions that consist on the conjugation of small units through a heteroatom linkage. Some reaction criteria should be respected which include, to be stereospecific, provision of high yields, simple isolation of the main product and mild reaction conditions [78]. Click reactions could be divided into two main types: alkyne–azide cycloaddition and thiol-ene/thiol-yne reaction [79]. The alkyne–azide cycloaddition is one of the most applied [80].

In the separation field, click chemistry was applied for the preparation of new CSPs being involved in the linkage of the chiral selector onto the chromatographic support and in the synthesis of new selectors [81]. During the immobilization of the chiral selector, it is possible to control the reaction conditions to prevent side reactions with non-desirable functional groups [79]. For example, to enhance the potential of cyclodextrin-based CSPs, click chemistry could be applied on the preparation of the called bridged cyclodextrin-based CSPs. Bridged cyclodextrins consist of two cavities linked through a chemical bridge, with different sizes and functional groups, which could establish additional interactions with the analyte [82]. The bridge also promotes an adjustment of the cyclodextrin contributing for a superior interaction with the target [82]. The advantages inherent to this type of CSPs are a remarkable binding capacity due to hydrophobic interactions and an improved stability of the complex [83]. The preparation of this type of CSP based on click chemistry proved to be a reliable alternative and selective methodology.

**Scheme 8:**
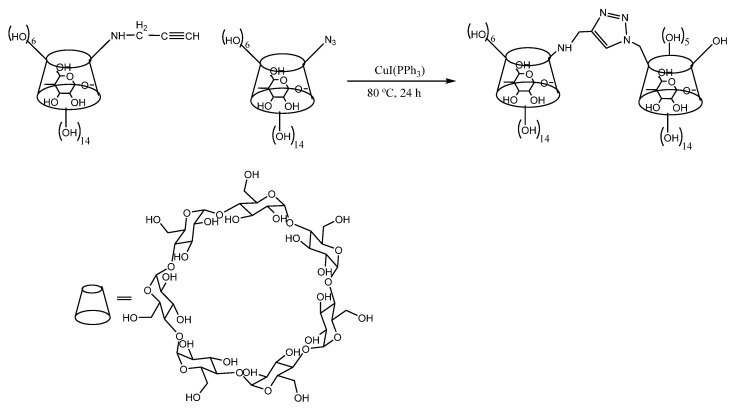
Synthesis of a triazole-bridged cyclodextrin-based chiral selector through click chemistry.

Shuang et al. [84] synthetized a triazole-bridged cyclodextrin-based CSP catalysed by copper (Scheme 8). Multi elution modes were explored and the new CSP presented an improved enantioselectivity, reproducibility and chemical stability than the native cyclodextrin CSP. The two cavities and the bridge interact with the target as a whole, promoting a synergetic effect which results from a broader separation range. This new CSP was characterized by FTIR, elemental analysis and TGA [84].

**Scheme 9:**
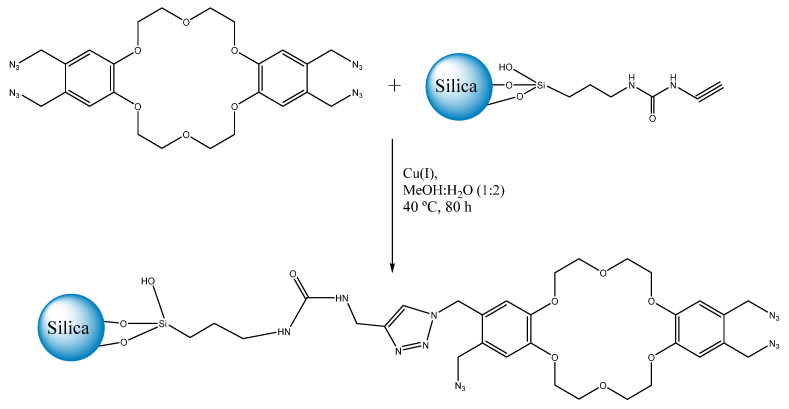
Synthesis of a crown-ether-based chiral stationary phase bonded to silica beads through click chemistry.

Li et al. [85] prepared a crown-ether-based CSP bonding the selector onto silica beads through click chemistry catalysed by copper (Scheme 9). The obtained CSP demonstrated toughness, chemical stability, and a satisfactory chromatographic performance under different elution modes. The click reaction proved to be an efficient methodology to link the chiral selector to the chromatographic support. CSP characterization was performed by FTIR and elemental analysis [85].

**Scheme 10:**
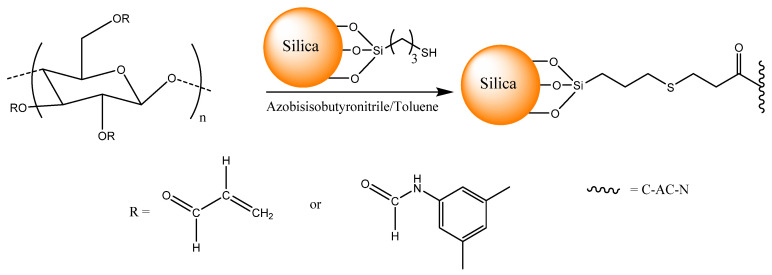
Synthesis of a cellulose-based chiral stationary phase through click chemistry.

Another example was described by Yin et al. [86], who prepared a cellulose-based CSP after chemical bonding onto thiol-modified silica gel (Scheme 10). The mixed esters of cellulose were chemically bonded onto the modified silica gel through a thiol-ene click reaction. The obtained CSP exhibited an increased resistance to organic solvents. The immobilization degree was controlled through the amount of acrylate groups introduced into cellulose chains to obtain cellulose mixed esters. A greater enantiorecognition was observed when compared with the coated cellulose *tris*(3,5-dimethylphenylcarbamte) CSP. For CSP characterization, FTIR and NMR analyses were performed [86].

**Figure 6:**
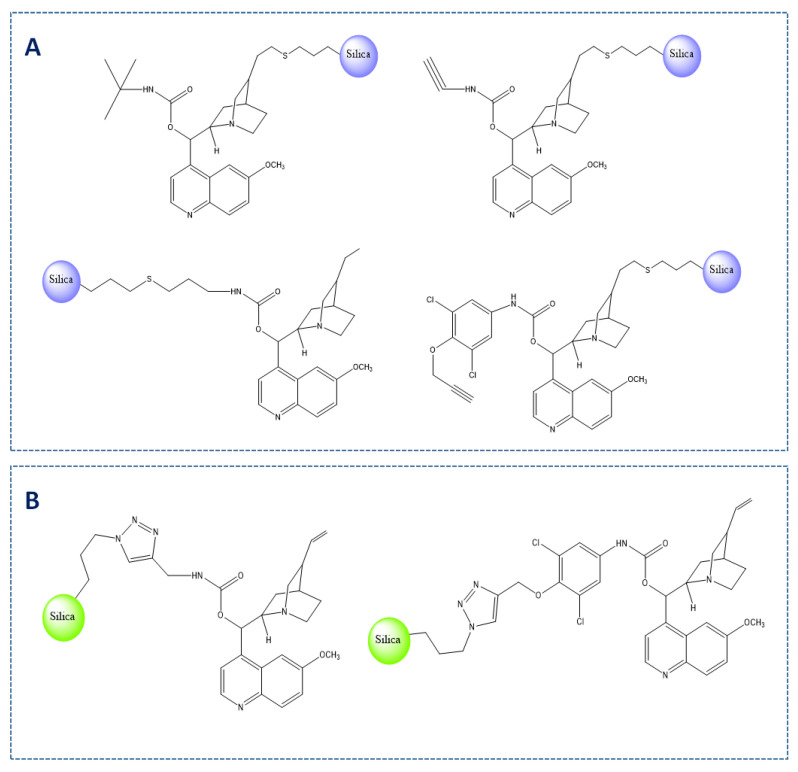
Anion-exchange-based chiral stationary phases synthetized through thiol-ene (**A**) and alkyne–azide (**B**) reactions.

Lindner et col. [87] compared the performance of anion-exchanger-based CSPs prepared by thiol-ene (Figure 6A) and copper-catalysed alkyne–azide (Figure 6B) reactions. The immobilization process performed by the thiol-ene reaction was applied to the double bond of the cinchona alkaloid while the alkyne–azide reaction was applied to the triple bond of the carbamoyl moiety [87]. 

CSPs presented high retention factors and enantioselectivity which could be explained by an enhanced interaction with the silica and the linker [87]. For the CSPs obtained by the thiol-ene process, a lower loading selector was preferred to achieve a superior selectivity and efficiency. An improved chromatographic performance was observed when compared with CHIRALPAK QD-AX^®^ and QN-AX^®^ columns. CSPs were characterized by NMR and elemental analysis [87].

### 3.6. Photochemical Method

In order to simplify the immobilization process, a photochemical method was introduced by Francotte [88], being motivated by the insolubility of chiral selectors on some organic solvents. The advantages inherent to this method are the simple procedure and no need of extra steps of protection and deprotection of functional groups, since no additional functional group was introduced on the chiral selector [88].

Accordingly, Francotte et al. [89] reported the preparation of a photochemically immobilized 4-methylbenzoyl cellulose-based CSP. The selector previously coated onto the chromatographic support was irradiated with UV light to promote the immobilization onto silica gel. The CSP combines a high chiral recognition capacity with a tolerance to a wide variety of organic solvents as mobile phases. An elemental analysis, NMR, FTIR and Raman spectroscopy were performed for its characterization [89].

**Figure 7:**
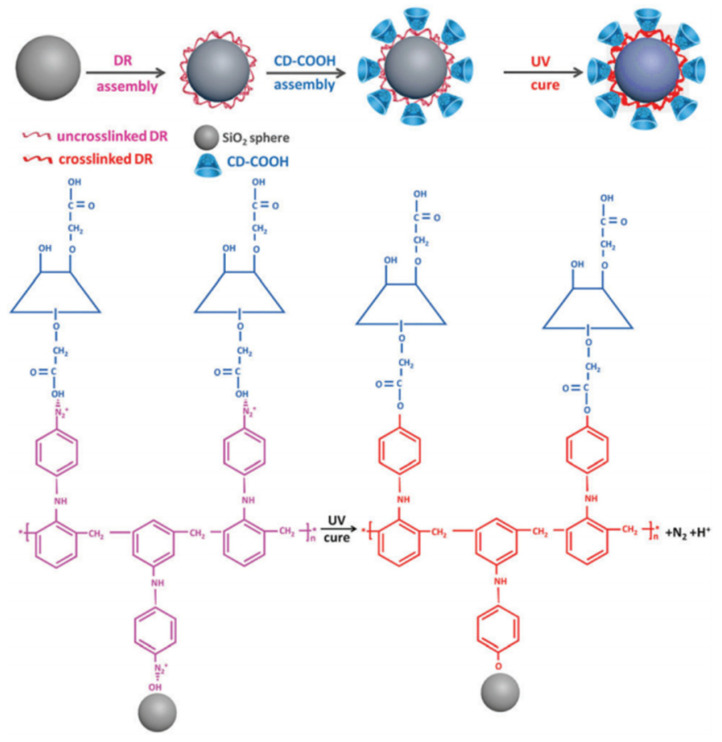
Light-assisted preparation of a cyclodextrin-based chiral stationary phase (reprinted from Publication Tang, Q.; Yu, B.; Gao, L.; Cong, H.; Zhang, S., Light-assisted preparation of cyclodextrin-based chiral stationary phase and its separation performance in liquid chromatography. *New J. Chem*. 2017, 42, (2), 1115–1120. Copyright © 2018, with permission from Royal Society of Chemistry [90]).

Another example is the light-assisted preparation of a carboxyl methyl β-cyclodextrin-based CSP (Figure 7), described by Tang et al. [90] using UV light to link the chiral selector to silica and to promote the modification of ionic bonds into covalent bonds. This technique proved to be environmentally friendly and efficient. The performance was evaluated under reversed-phase and polar organic elution mode demonstrating enantioselectivity potential. CSP characterization was performed using FTIR, TGA and SEM [90].

A vancomycin-based CSP was recently prepared by Yu et al. [91] using the same method. After the treatment with UV light, the ionic bonding was converted into covalent bonding through a photochemical reaction. Different elution modes were explored to evaluate its performance, showing a promising chiral resolution for a variety of chiral analytes. SEM, FTIR, NMR and TGA were applied for the characterization of this CSP [91].

### 3.7. Thermal Method

Concerning the reduction in chemical reagents used during the immobilization process, a thermal method was introduced by Francotte [92], in 1997. As in the photochemical method, the chiral selector should be previously coated onto the chromatographic support before the immobilization.

**Figure 8:**
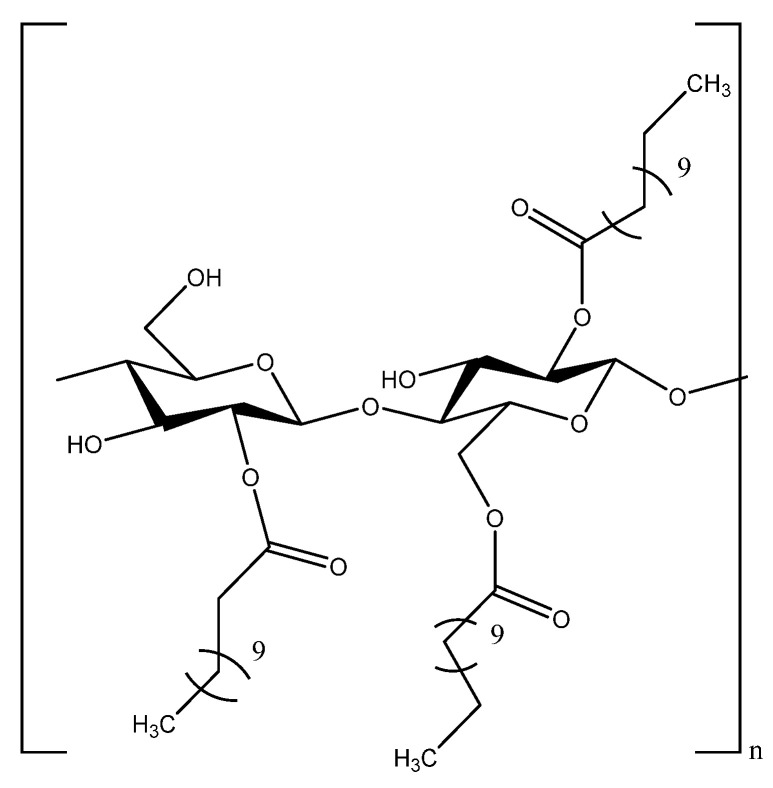
Cellulose dodecanoate selector for thermal immobilization on silica.

A recent example was reported by Vieira et al. [93] that described the immobilization of cellulose dodecanoate onto silica particles at a high temperature and without a chemical agent (Figure 8). It was found out that the particles retained the spherical form and no agglomeration was observed. The strong retention of the selector onto the chromatographic support and the reduced cost of this environmentally friendly methodology were emphasized. The characterization of the CSP was performed by an elemental analysis, FTIR, SEM and nitrogen adsorption isotherms with BET and TGA [93].

## 4. Summary of the Characterization Methods of CSPs

After the preparation of a CSP, it is fundamental to proceed with the characterization of the CSP. The characterization step is important since the physicochemical properties, the morphology and load affect the chromatographic parameters, such as the retention factor, enantioseparation, resolution, number of theoretical plates, column backpressure and stability of the CSP [94]. Moreover, the presence of morphological irregularities can also interfere with the backpressure and the diffusion pattern [95].

As demonstrated by all the examples previously mentioned, CSP characterization could involve several techniques. Table 1 summarizes the main techniques according with the property to be evaluated, physical or chemical. Almost all the mentioned techniques, can be used in combination allowing a complete analysis and characterization of the CSPs.

## 5. Conclusions

To follow the constant challenges on different areas as well as the advances in the chromatographic instrumentation and technical progress, the development of new CSPs for LC has been a continuous and evolutionary subject. Recent reports related with the introduction of new strategies for the preparation of CSPs were presented. The referred strategies comprised modifications of the traditional coating technique and the introduction of new methods for immobilization onto chromatographic supports. The main focus was to obtain new CSPs with an increased efficiency, stability and cost-effectiveness as well as the introduction of more environmentally friendly approaches. Some of the implemented strategies allowed the possibility to use non-conventional solvents as mobile phases, in coated-type CSPs, resorting to polymeric coatings. An improved resistance to a wide range of pH values, compared with already existing CSPs, was also focused on being introduced into coatings on inorganic oxides. A dynamic coating was also revolutionized being the only in situ technique described. Principles of green chemistry sustained some works, which also motivated the introduction of some new strategies. In this topic, it is important to highlight the immobilization based on the click chemistry, photochemical and thermal methods.

Despite the selection of the most appropriate chiral selector as well as the chromatographic support, the choice of the technique applied for the preparation of the CSP could compromise the chromatographic performance. It is crucial to decide on the most suitable technique for preparation (coating or immobilization) according with the chiral selector and the final application. In a future perspective, we believe that the classical and broadly applied coating and immobilization procedures will continue to be described for the preparation of new CSPs. Nonetheless, the trend will be to explore in a more meaningful way the innovating strategies herein presented. Moreover, considering that, recently, new types of materials are being introduced as chromatographic supports and/or chiral selectors (monoliths, metal–organic frameworks, covalent–organic frameworks, among others), our opinion is that new synthetic strategies will be designed for allowing the development of the desired CSPs.

The characterization of the products for the packing column is also an important aspect of this area of research, as the degree of coating and/or immobilization and the morphology of material significantly affect the chromatographic parameters. According to the examples reported, the main techniques applied include NMR, TGA, FTIR and elemental analysis.

The preparation of diverse CSPs using different strategies is an important trigger aiming to achieve new CSPs with a high versatility and an extended range of analytical and preparative applications. The development of more efficient chromatographic tools for enantioselective LC has beneficial outcomes in a large variety of areas.

## Figures and Tables

**Table 1:** Techniques for characterization of physical and chemical properties.

Characterization of Chemical Properties		Characterization of Physical Properties	
Technique	Parameter	Technique	Parameter
Elemental analysis	Loading/degree of derivatization	SEM	Particle morphology and size
FTIR	Identification of functional groups/derivatization pattern	N_2_ or argon adsorption isotherms at the temperature of liquid nitrogen in conjunction with BET theory	Surface area Pore volume
Inductively coupled plasma-atomic emission spectrometry (ICP-AES)	Determination of metallic impurities in silica or modified silica materials	Low-angle powder X-ray diffraction (XRD)	Pore size
NMR	Structure elucidation of organic and inorganic compounds	Laser particle sizer analysers	Particle size
TGA	Determination of silanol groups quantity, quantification of organic groups presented and estimation of thermal stability	Combination of XRD data and average pore diameter	Wall thickness
Diffuse reflectance infrared Fourier-transform spectrometry (DRIFTS)	Evaluation of silanol density and water adsorption on silica surface	TEM	Pore size

## Data Availability

Not applicable.
